# A general approach to detecting migration events in digital trace data

**DOI:** 10.1371/journal.pone.0239408

**Published:** 2020-10-02

**Authors:** Guanghua Chi, Fengyang Lin, Guangqing Chi, Joshua Blumenstock

**Affiliations:** 1 School of Information, University of California, Berkeley, Berkeley, CA, United States of America; 2 Department of Statistics, Columbia University, New York, NY, United States of America; 3 Department of Agricultural Economics, Sociology and Education, Pennsylvania State University, University Park, PA, United States of America; University of Wisconsin Madison, UNITED STATES

## Abstract

Empirical research on migration has historically been fraught with measurement challenges. Recently, the increasing ubiquity of digital trace data—from mobile phones, social media, and related sources of ‘big data’—has created new opportunities for the quantitative analysis of migration. However, most existing work relies on relatively *ad hoc* methods for inferring migration. Here, we develop and validate a novel and general approach to detecting migration events in trace data. We benchmark this method using two different trace datasets: four years of mobile phone metadata from a single country’s monopoly operator, and three years of geo-tagged Twitter data. The novel measures more accurately reflect human understanding and evaluation of migration events, and further provide more granular insight into migration spells and types than what are captured in standard survey instruments.

## Introduction

Migrants play an important role in all aspects of modern society. It is estimated that about 0.6% of the world population migrated internationally from 2005 to 2010 [1]. As many as 750 million people in the developing world are permanent internal migrants [2]. Understanding the causes and effects of migration is a central focus of social science research. For research and policy, it is thus critical to have an accurate quantitative understanding of the scale and scope of migration.

However, empirical research on migration has historically been hindered by the lack of granular migration data. Traditional data on migration are typically derived from population censuses or sample surveys, and are usually based on questions about place of birth and recent migrations. But census and surveys are expensive and time-consuming, and are plagued by issues of attrition since migrants, by definition, do not remain in the same place [2, 3].

Over the past decade, the mass proliferation of digital devices has created large repositories of ‘digital trace’ data, which provide new opportunities to measure and model human mobility. The data most commonly used in such studies are collected by mobile phone networks or social media platforms. While the majority of such studies focus on local mobility [4–6], several more recent papers have used such data to analyze migration [7–9]. In turn, migrant flows have been used to study labor markets [10–12], infectious diseases [13–15], disaster response [16, 17], and other social phenomena linked to migration.

**Fig 1:**
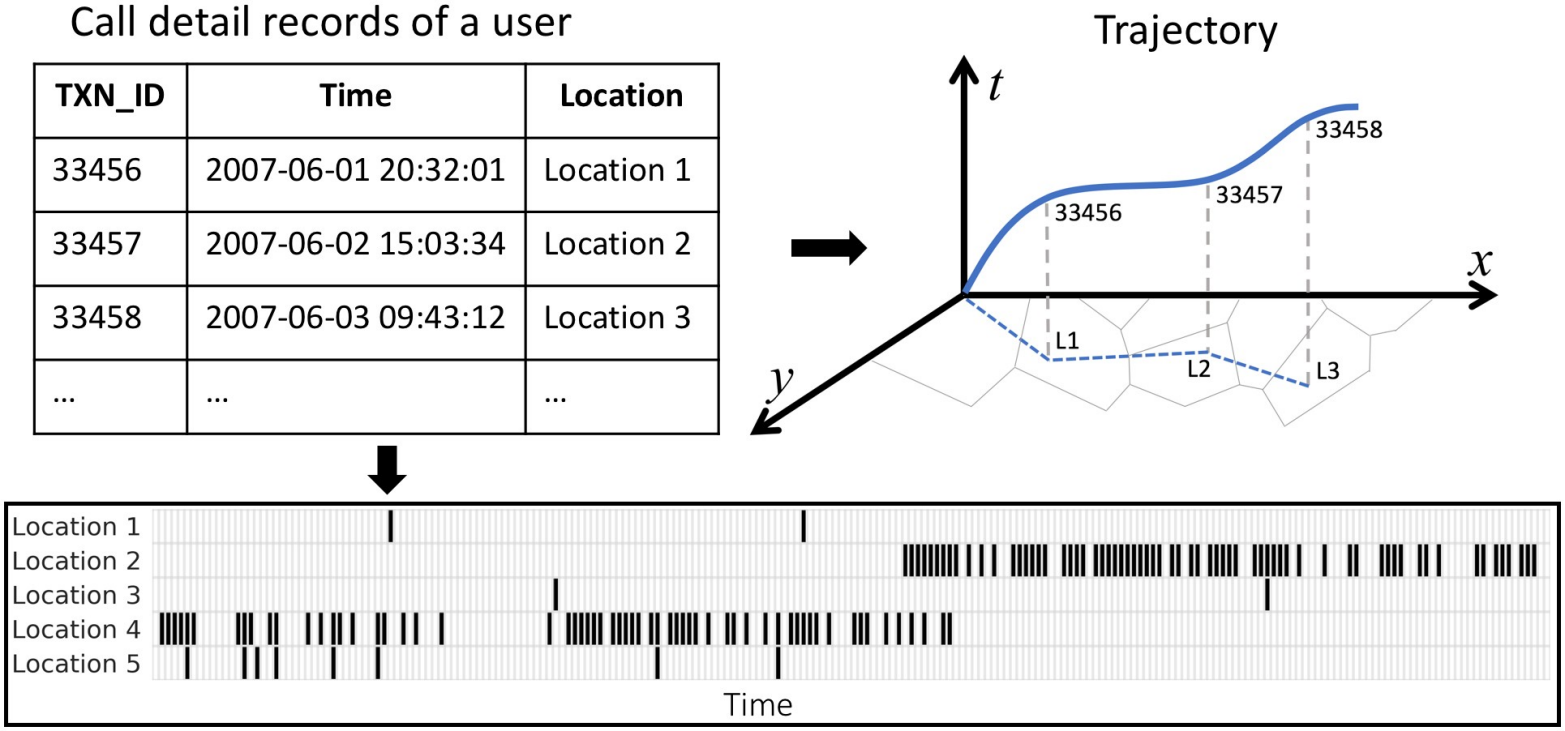
Extracting human trajectories from trace data. Raw data (*top left*) contains timestamps and geo-coordinates each time each individual is active on the platform (e.g., making a phone call). From these data, the trajectory of the person through space and time can be reconstructed (*top right*). The bottom figure shows the set of locations (e.g., neighborhoods) in which the individual was observed on each day.

A stylized representation of these data, and how they can be used to reconstruct human trajectories, is shown in Fig 1. The top-left table shows the ‘raw’ data that is logged by, for instance, a mobile phone operator. These transactions can be mapped to physical locations, which indicate the person’s trajectory (top right map). We will also use two-dimensional arrays (bottom figure) to visualize location decisions over a long time horizon.

Most existing studies of migration based on trace data rely on relatively *ad hoc* methods for measuring migration. Prior work typically breaks the problem down into two distinct steps: First, the data are used to infer the “home location” of an individual at a certain point in time; and second, the sequence of home locations is used to infer migration events. This approach, while simple, has several important limitations. For instance, most studies make the assumption that an individual’s ‘home’ location is the cell tower or the city from which they make the most of the calls or post the most tweets in a defined time period. For example, Zagheni et al. [8] and Fiorio et al. [18] assign users to the county from which they posted the majority of tweets during a specified period of time. Papers using phone data typically assign home locations based on the cell tower or administrative unit with the densest call activities [7, 9, 19–21]. See S1 Table for a full inventory of the prior work using trace data to study migration.

**Fig 2:**
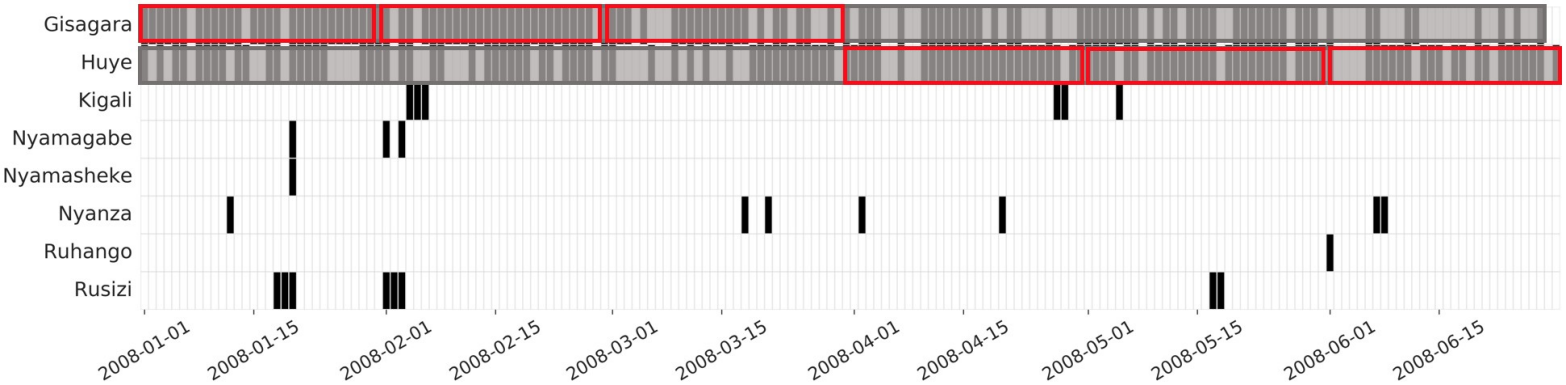
One individual’s locations over six months. Each row is a different district, each column is a day; cells are colored black if the individual either made or received a call in that district on that day. The red boxes show the individual’s modal district in each month.

But such inferences are not robust to the bursty behavior that has been well-documented in phone and media use [22]. More generally, this notion of ‘home’ is brittle to diurnal patterns (e.g., home vs. work device use) and the measurement technology used (for instance, the fact that phone networks load balance by shifting calls from high-volume cell towers to neighboring low-volume cell towers). An example of how such inferences can go wrong is shown in Fig 2, which shows the different locations in which a single mobile phone subscriber was observed over a 6-month period in Rwanda. In the case, the individual’s most frequent location for the first three months was different from the most frequent location for the last three months, but in reality the individual simply lived in the border region between the two locations, and did not actually migrate (the modal cell tower of this mobile phone subscriber in the first three months is roughly 500 meters from the border with Huye).

To fill these gaps in the literature and enable future empirical work on migration, this paper develops a novel and general approach to detecting migration events in large-scale digital trace data. We begin by proposing a new segment-based algorithm for migration detection. This algorithm first groups contiguous segments in time when an individual is likely to be in the same location (subject to some random deviations), and then identifies persistent changes in those segments over time. The algorithm is intuitive, and contains tuning parameters that make it possible to flexibly detect both short-term displacement and long-term migration. It also makes it possible to identify the likely date of migration, and provides a confidence intervals for each inferred migration event.

We then conduct a series of experiments to calibrate and validate this new algorithm using mobile phone and Twitter data. In particular, we hired a team of students to hand-label 1,000 migration diagrams (see S1 Fig), and compared these hand labels to our algorithm’s predictions, and to those of alternative methods in the literature. We show that our approach is substantially more accurate than traditional approaches.

By providing a more coherent and robust framework for detecting migration, we hope this study can improve the set of tools available to applied researchers, and in turn advance empirical research on migration. The method we develop is ‘data-agnostic’ in the sense that it can be applied to any dataset where individuals have spatial and temporal markers, and no other contextual information is available. In cases where the specific digital trace dataset contains more detailed information (such as message content or social network structure), or where contextual information is available (such as information on places of interest), more sophisticated, custom analysis methods may improve location inference [19, 23, 24]. To facilitate adoption by the research and policy community, we have packaged the algorithms into a set of tools that are implemented using an open-source Python-based library (available at https://github.com/g-chi/migration_detector).

## Background and related work

Empirical analysis of human migration dates back at least to 1885, when Ravenstein analyzed 1881 British census data with the information of birthplace and residence place [25]. The research was done at a time of a large number of migrants after the Second Industrial Revolution [26]. Ravenstein summarized seven “laws of migration”, such as “the great body of our migrants only proceed a short distance, and that there takes place consequently a universal shifting or displacement of the population” [27, 28].

More recently, the research literature has defined a migration event as “a change in the place of usual residence, which also involves crossing a recognized political/administrative border” [29]. In practice, this usually involves specifying a temporal dimension and a spatial dimension [30, 31]. The temporal dimension indicates some fixed length of time in which an individual must remain in a location for residency to be established; the spatial dimension typically involves crossing international or internal administrative boundaries [32]. For instance, the US Census asks the residence of households in the previous year and the year when the households moved to the current residence. The World Bank’s Livings Standards and Measurements Survey, conducted primarily in developing countries, similarly contains a migration module that queries place of birth, the year that households moved into the current housing unit, and other relevant information [29, 33].

Over the past decade, a handful of studies have used novel sources of spatiotemporal ‘trace’ data to observe human mobility and migration with much greater spatial and temporal granularity. We think of ‘trace’ data as that produced as the result of people’s ordinary activities that leave behind a digital footprint, rather than data produced specifically for the purpose of scientific study [34]. *Spatiotemporal* trace data contain spatial and temporal markers. Early research in this area used mobile phone data to characterize patterns of human mobility. For instance, González et al. [4] show that human mobility is highly regular and follows a truncated power-law distribution. Song et al. [5] similarly find that human mobility is highly predictable, and others develop statistical models of human mobility [35–48].

More recent work has used digital trace data to study human migration. This body of work, and the way the data are used to measure migration, are summarized in S1 Table. In work most closely related to the current study, Blumenstock [7] proposed a rudimentary method for inferring migration from phone data, which defined a migration event as one in which an individual remains within one administrative unit for *k* consecutive months and then a different administrative unit for *k* consecutive months. This approach, and its slight variations, have subsequently been used to study migration using phone data [10, 21, 36] and social media data [8].

An important related body of work grapples with the ethical considerations inherent in using digital trace data to study human behavior in general [49, 50], and the privacy concerns that arise in using such data to study human mobility specifically [51, 52]. The methods we describe in this paper are meant to provide more accurate and robust measurements of human migration, and while our goal is to enable social science research and pro-social applications (e.g., rapid disaster response), we acknowledge the potential for anti-social uses (e.g., discrimination against at-risk populations). The analysis we conduct here uses de-identified data and is governed by strict IRB protocols; we can only urge subsequent applications and extensions to use these methods and data responsibly.

This paper contributes to the literature by developing and validating a more robust approach to inferring migration from spatiotemporal trace data. In the next section, we describe this approach and show how it can be used to identify migrants, infer migration dates, and provide measures of confidence for each migration event. We then walk through a series of examples to demonstrate how the algorithm works on data. Last we calibrate and validate the algorithm by comparing the algorithm’s predictions to those of human judges and alternative approaches.

## Detecting migration: A 3-step algorithm

In this study, we define a migration event as one where an individual’s primary residential location remains stable for some minimum amount of time, and then changes to a different location for another minimum amount of time. Following Blumenstock [7], we define a ‘minimum amount of time’ flexibly, using a parameter *k* that can be easily changed to suit the application domain. While most of our empirical examples fix *k* = 3 months, longer-term migrations and shorter-term displacements could be studied analogously by increasing or decreasing *k*. We allow ‘locations’ to be flexibly defined as any pre-existing administrative division. In the empirical examples that follow, we use administrative districts in Rwanda (in the mobile phone data example), and cities in the United States (in the Twitter data example).

Using this empirical definition of “migration”, a key contribution of this paper is to design an algorithm that operationalizes this definition on digital trace data. The algorithm, which is included as S1 Algorithm, operates in three steps: First, it detects contiguous location segments in the raw trace data. Then, it determines which of those segments constitute ‘migration’ events. Finally, it infers an exact migration data, and assigns a confidence interval to each migration event.

### Detecting location segments

**Fig 3:**
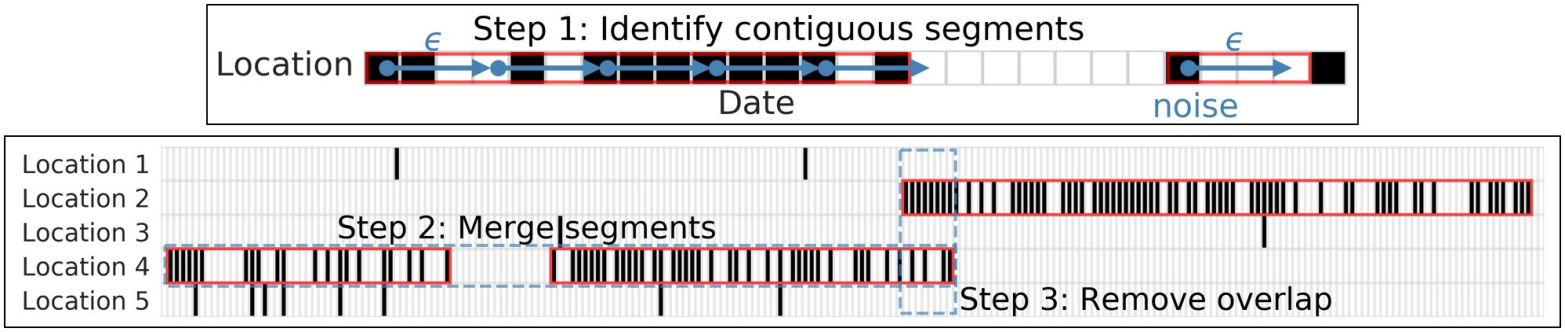
Detecting location segments. Step 1 identifies segments where an individual is at a location continuously, with no gaps exceeding *ϵ* days. Red boxes in the bottom figure are detected segments. Step 2 merges neighboring segments together. Step 3 removes overlap between two segments.

The first step in detecting migration requires detecting periods of time when an individual is continuously present in a single location, allowing for some margin of travel from that location (for instance, for an evening on the other side of the city, or for a weekend trip out of town). We accomplish this in 3 substeps. A schematic of this process is depicted in Fig 3. The full details of this algorithm are given in S1 Algorithm, and summarized below.

- Step 1: Identify contiguous segments. The goal of this step is to identify contiguous periods of time during which a person remains in the same location. For simplicity, we assume the raw latitude/longitude coordinates can be resolved to locations with preexisting administrative boundaries (such as neighborhoods or municipalities). We use a clustering algorithm, similar to DBSCAN [53], that finds periods when an individual remains at a single location continuously, with no gap exceeding *ϵ* days. To allow for idiosyncratic deviations, from a primary location, we consider all segments where the individual is observed in that location on at least *propDays* percent of days in the segment. Finally, we eliminate segments that are less than *minDays* days in length.
- Step 2: Merge segments. This step merges neighboring segments together if there are no segments in other locations between them.
- Step 3: Remove overlap. This step resolves situations when an individual is associated with segments in multiple locations at a single point in time.

### Detecting migration

After contiguous location segments are identified, migration events are defined by the existence of two neighboring segments with different locations. This requires specifying a minimum residency *k* for the individual to be in each location. As there is no universal definition of residency length [32], we expect different applications to use different values for *k*. In the empirical examples below, we use *k* = 90 days. The efficiency of our algorithm is *O*(*n*), where *n* is the number of records in a trajectory dataset.

### Inferring the date of migration

We also design a method to infer the exact date on which a migration occurs, rather than, say, simply recording that a person was in one location in one month and a different location in a subsequent month. In cases where there is a discontinuous break such that an individual appears only at one location until a specific day *t*, and then only at a different location after that day, then we simply say that the person migrated on day *t*. Often, however, there is some ambiguity, such that an individual is observed in both one location and another, and the exact migration date is not obvious (as in the dotted blue region of Fig 3). In such cases, we select the day between the start of the new segment and the end of the old segment that minimizes the number of ‘misclassified’ days, i.e., the number of days when the migrant appears at destination before the migration date and days when the migrant appears at home after the migration date. In cases where multiple days yield the same number of misclassifications, we select the last day as the migration date.

### Measuring the uncertainty of migration dates

For every migration date detected through the above algorithm, we attach a measure of confidence that an actual migration occurred. Confidence measures are useful because, as can be seen in Fig 2, the raw data are often quite noisy (and occasionally very sparse), and some migration events are more ambiguous than others. To evaluate the uncertainty of the inferred migration dates, we use the number of gap days between the start of the new segment and the end of the old segment after removing overlap. The larger the gap is, the higher uncertain our estimation is.

## Empirical example

**Fig 4:**
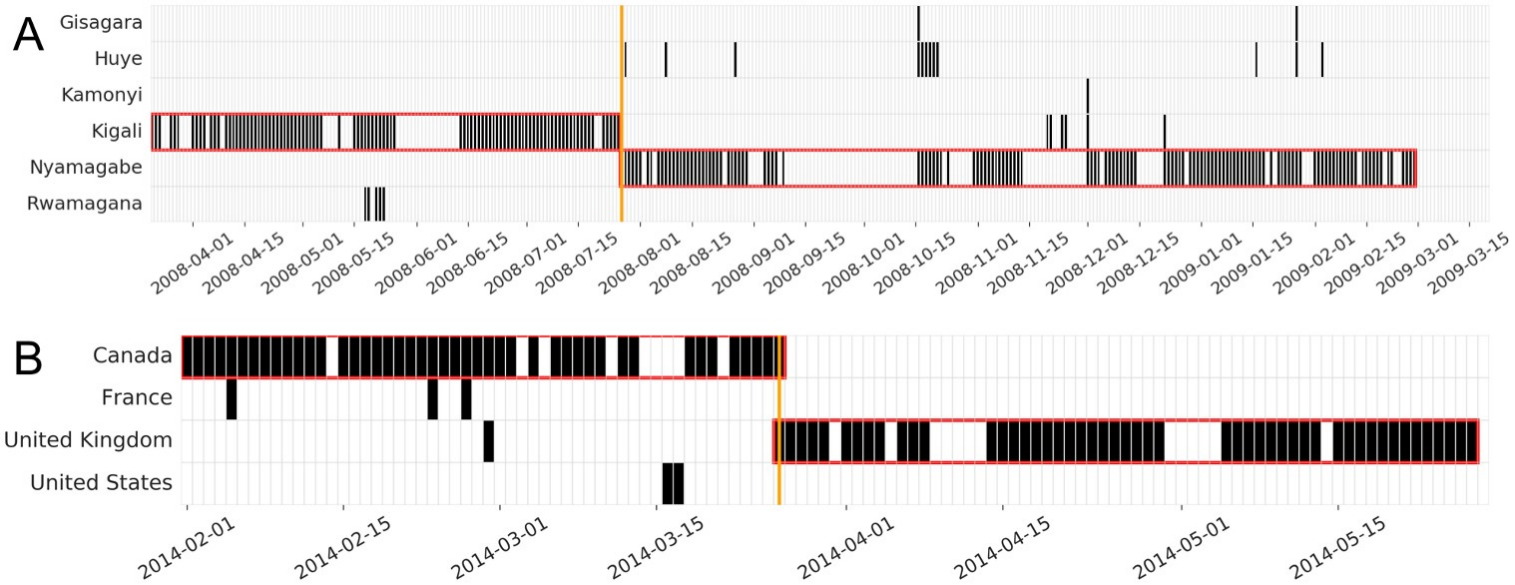
Trajectories of two migration events detected by our approach. Each row is a different district, each column is a day; cells are black if the individual makes or receives a phone call from that district on that day. Red boxes indicate location segments. Orange line marks the inferred date of migration. (A) Long-term migration in Rwanda (migrated from Kigali to Nyamagabe on 2008-07-27). (B) International migration (migrated from Canada to United Kingdom on 2014-03-26).

Above, we describe a general algorithm for detecting migration events in trace data. To provide some intuition for how this algorithm works in practice, we show the results when applied to two different sequences of location data. Fig 4 displays the location history, location segmentation, and inferred migration dates from one mobile phone trace and one Twitter trace. Fig 4A shows the trajectory of a long-term migrant from Kigali to Nyamagabe. Even though some noise exists in this individual’s trace data (i.e., they are seen in multiple locations and there are many days without any trace data), the method identifies a migration event. Note that in this example, the standard approach of first identifying primary locations in each month and then inferring migrations would fail, since the modal location in October 2008 is Huye rather than Nyamagabe. Fig 4B shows the trajectory of an international migrant who moved from Canada to the United Kingdom. Compared to within-country migration using mobile phone trace data, international migration detected on the basis of geo-tagged tweets has less noise and is relatively easier to detect segments.

## Experiments and validation

The preceding examples provide some intuition for how the algorithm works when applied to data. To provide more rigorous quantitative validation of the method, we run a series of experiments with human judges. This makes it possible to compare the performance of this algorithm to traditional approaches, highlight the overall robustness of this method, and show how the algorithm’s parameters can be tuned.

### Traditional frequency-based methods

Frequency-based methods in most prior work first assign individuals to locations in each month (based on the location in which the majority of events occur in that month) and then classify migrants as individuals whose locations change between subsequent months (S1 Table). There are different methods to estimate home locations in each month. Vanhoof et al. [54] summarized five different methods of detecting home locations: (method 1) the location with the maximum number of phone activities; (method 2) the location with the maximum number of distinct days with phone activities; (method 3) the location with the maximum number of phone activities during 7 p.m. and 9 a.m.; (method 4) similar to the method 1, but aggregating all activities within a spatial perimeter of 1,000 meters around a cell tower instead of counting phone activities only in each cell tower; (method 5) similar to the method 4, but only counting phone activities recorded during 7 p.m. and 9 a.m. A hierarchical method, proposed by Blumenstock et al. [10], which identifies locations where the individual spends the most time, is method 6. These six frequency-based algorithms are implemented using Python (available at https://github.com/g-chi/migration_detector/blob/master/frequency_based_method.py).

Specifically, the method in Blumenstock et al. [10] first identifies the *hourly* modal location by computing the most frequently visited district in every hour of the entire dataset, then aggregates hourly modal locations to find the *daily* modal location, and finally identifies the *monthly* modal location by taking the mode over the daily modal locations.

### Experimental design

#### Digital trace data

To help illustrate the generality of our approach, we perform experiments on two different digital trace datasets.

- **Mobile phone data**. We obtained a pseudonymized dataset of mobile phone activity directly from Rwanda’s near monopoly mobile phone operator. These data contain the complete Call Detail Records (CDR) of roughly 1.5 million de-identified individuals, covering a period of 4.5 years. Each time a mobile phone owner makes or receives a call or text message, a new entry is generated in this dataset which contains a unique identifier for the caller and receiver, a timestamp for the event, and the approximate location of the caller and receiver (i.e., the geo-coordinates of the nearest cell phone tower).
- **Twitter data**. The geo-tagged tweets we use include 20,000 randomly selected Twitter users in the U.S. over two years who have at least 1,000 geo-tagged tweets. Each record in this data contains a unique identifier for the individual, the timestamp of the tweet, and the geo-coordinates from which the tweet was posted.

Note that the key commonality between these two datasets—and what is required for our algorithm to work—is that a single transaction record in both datasets contains spatial and temporal information. This research is exempt from IRB approval because we use only de-identified data.

#### Validation data

To validate our approach, we hired a team of five undergraduate students to build a labeled corpus of migration data. Each labeler was randomly assigned a large number of ‘samples’, where each sample contains a trajectory for a single individual (see S1 Fig for an example). Each sample was judged by three labelers. If three labelers had different opinions on one sample whether it is a migrant or not, we chose the label that was agreed by at least two labelers. For each sample, the human labeler was required to indicate (i) whether a migration took place; (ii) how confident they are in that assessment on a scale of 1 to 3. In addition, if a migration was marked, the labeler was asked (iii) the date of migration; (iv) their confidence in that date at an interval of 5 days (0-5, 6-10, 11-15, >15); (v) the first day and last day of home segment and destination segment.

In total, the labelers provided labels for 1,000 different migration trajectories. These 1,000 samples were drawn strategically to compare and contrast our new *segment-based* approach and the ‘traditional’ *frequency-based* approach used in most prior work. Specifically, we drew: (1) 250 samples where both algorithms detect a single migration; (2) 250 samples where neither algorithm detects a migration; (3) 250 samples where the new method detects a migration but the traditional method does not; and (4) 250 samples where the traditional method detects a migration but the new method does not.

### Experimental results

We compare the performance of the new method of detecting migration to the traditional method in Table 1 using four criteria: accuracy ($\frac{True\;positive+True\;negative}{all\;samples}$), precision ($\frac{True\;positive}{True\;positive+False\;positive}$), recall ($\frac{True\;positive}{True\;positive+False\;negative}$), and F1 score ($2*\frac{precision*recall}{precision+recall}$). The accuracy of the six frequency-based methods ranges from 61.3% to 70.6%. The best frequency-based method is method 2, which defines the home as the location with the maximum number of distinct days with phone activities. This result also confirms the findings by Vanhoof et al. [54], who compared the first five methods based on a 6-month CDR dataset from France and found that method 2 has the best performance. By comparison, our proposed algorithm obtains an accuracy of 84.5%, with both higher precision and recall than common frequency-based alternatives.

**Table 1:** Performance of the six frequency-based algorithms and our proposed segment-based algorithm. See section “Traditional frequency-based methods” for the details of the six frequency-based methods.

Method	Accuracy	Precision	Recall	F1
Frequency-based 1	0.644	0.584	0.619	0.601
Frequency-based 2	0.706	0.633	0.762	0.692
Frequency-based 3	0.692	0.649	0.628	0.638
Frequency-based 4	0.677	0.617	0.670	0.642
Frequency-based 5	0.693	0.651	0.628	0.639
Frequency-based 6	0.613	0.546	0.630	0.585
Segment-based	0.845	0.778	0.898	0.834

Since many of the samples in the dataset are ambiguous (as in S2 Fig), we asked labelers to indicate their own level of confidence in classifying the sample as a migration or non-migration. Importantly, we find that the segment-based method performs better than the frequency-based method irrespective of the ambiguity of the underlying sample. This result can be seen in Table 2, which disaggregates the performance of each algorithm by the (human-classified) ambiguity of the sample. The new method is more accurate than the traditional method for each of the three types of samples. In the type of “somewhat confident”, our method is 18.3% more accurate than the traditional method. Note that for those cases where labelers have different opinions on whether a migration takes place in a sample, we only keep the results agreed by two labelers in this table. This is the reason why the total number of samples in the three categories of uncertainty is different for the two approaches.

**Table 2:** Performance of the two approaches at labelers’ different levels of confidence.

Method	Uncertainty by labelers	Accuracy	Precision	Recall	F1	# Samples
Frequency-based	overall	0.706	0.633	0.762	0.692	1000
	not confident at all	0.521	0.419	0.646	0.508	376
	somewhat confident	0.598	0.543	0.690	0.608	892
	very confident	0.810	0.762	0.827	0.793	1732
Segment-based	overall	0.845	0.778	0.898	0.834	1000
	not confident at all	0.566	0.452	0.625	0.525	376
	somewhat confident	0.781	0.713	0.864	0.781	892
	very confident	0.901	0.865	0.920	0.892	1732

Related, we find that when all judges concur on the classification of a given sample, the performance of all algorithms is higher, and that irrespective of the level of inter-judge agreement, the segment-based approach is more accurate. In cases when all judges give the sample the same label, the accuracy of the segment-based approach is 92.3% (and the accuracy of the best frequency-based method is 78.9%). In cases where not all judges agree, the accuracy of the segment-based approach falls to 79.5% (and the accuracy of the frequency-based method drops to 65.1%).

**Fig 5:**
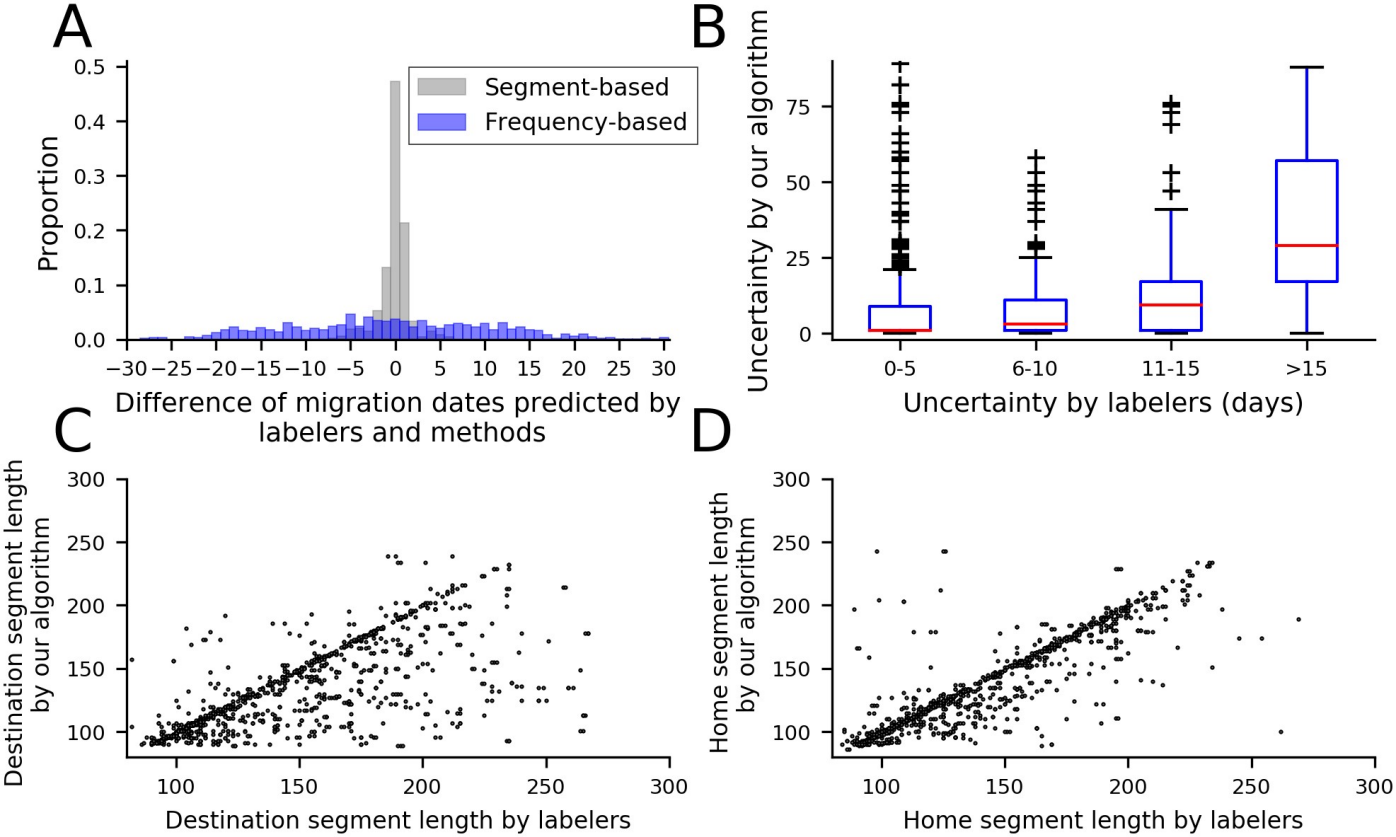
Performance of our approach. (A) Distribution of migration date difference between our approach and labelers. (B) Distribution of uncertainty between our approach and labelers. (C) Distribution of destination duration. (D) Distribution of home duration. Our method has high accuracy on the estimated migration dates and home and destination segment length.

Our method has high accuracy on the estimated migration dates and home and destination segment length. Fig 5A shows that most of the migrants have a very small difference between real migration dates and estimated migration dates. 77.67% of migration events have the error within two days based on our approach, while for the best frequency-based algorithm (method 2), 15.39% of migrants have the error within two days. The frequency-based algorithm only reaches comparable accuracy (76.73%) when the error margin is increased to 15 days. This implies that our approach has a good performance in estimating migration dates. Note that we do not know the true date of migration. Rather, the migration dates we assume to be true are those assigned by the five student labelers. To measure this uncertainty, we ask labelers their uncertainty on the migration dates. As described earlier, our algorithm associates a measure of confidence with each inferred migration date. We compare this measure of confidence/uncertainty to the level of uncertainty assigned by the human labeler, for the set of samples where the human attempted to assign a migration date to the sample. Fig 5B shows that the uncertainty estimated by our method visibly increases as human judges express uncertainty in their judgement on the date of the migration. Fig 5C and 5D confirm that our approach can also find reasonable residency length.

Why does the new segment-based approach out-perform the traditional frequency-based approach? One common (false positive) error of the traditional methods is to falsely identify as migrants people who frequently appear in neighboring locations (as in Fig 2). Another common (false negative) error of the traditional methods is to erroneously classify transient displacement as a change of primary location (as in Fig 4A), so that the person does not remain stable for long enough to be classified as a migrant. In both these situations, the segment-based approach performs better.

### Qualitative validation of Twitter samples

The main experiments described above validate our segment-based method using a large sample of mobile phone data. However, the algorithm we describe is much more general, and in principle can be applied to any digital trace dataset with spatial and temporal markers.

To better illustrate the generality of this method, we apply the segment-based algorithm to infer migration events from a small sample of Twitter. Specifically, we pull the Twitter histories of 100 individuals who the segment-based algorithm identified as migrants, and analyze the contents of their tweets immediately before and after the date of migration inferred by the algorithm. In 91% of these cases there is direct evidence in the contents of the tweets that a migration indeed occurred on the inferred date. To provide one example, Table 3 contains the tweets posted by one individual in the time surrounding migration. The algorithm infers a migration date of September 4, which is consistent with the text of the tweets.

**Table 3:** Selected tweets of a detected migrant who moved from Virginia to New York on 2014-09-04 based on our approach.

Date	Tweet
2014-08-28	hmm. second to last day in Charlottesville! #daydreamin
2014-08-29	Moving out of Cville today #ahhhh
2014-09-04	Walking around Union Square with this crazy beautiful weather got me like whoa #nyc
2014-09-07	What else to do in nyc when it’s 85 and gorgeous?? Go to the beach! #forttilden

We compare the performance of the frequency-based approach to our segment-based approach using Twitter data. We find that the frequency-based approach detects more “false positives”—i.e., non-migrants who appear to be migrants because they have sparse trajectories. Using the frequency-based method, only 57.7% of days are attributed to either the home or destination district; this proportion rises to 72.9% with the segment-based method.

As several prior studies have pointed out, there are large biases in Twitter use (including who enables geolocation information on their Tweets) that complicate the interpretation of migration statistics derived from Twitter data (cf. [8, 18, 55, 56]). Our tests with Twitter data are primarily intended to illustrate the potential of our algorithm to be applied to an arbitrary source of digital trace data.

### Tuning algorithm parameters

**Fig 6:**
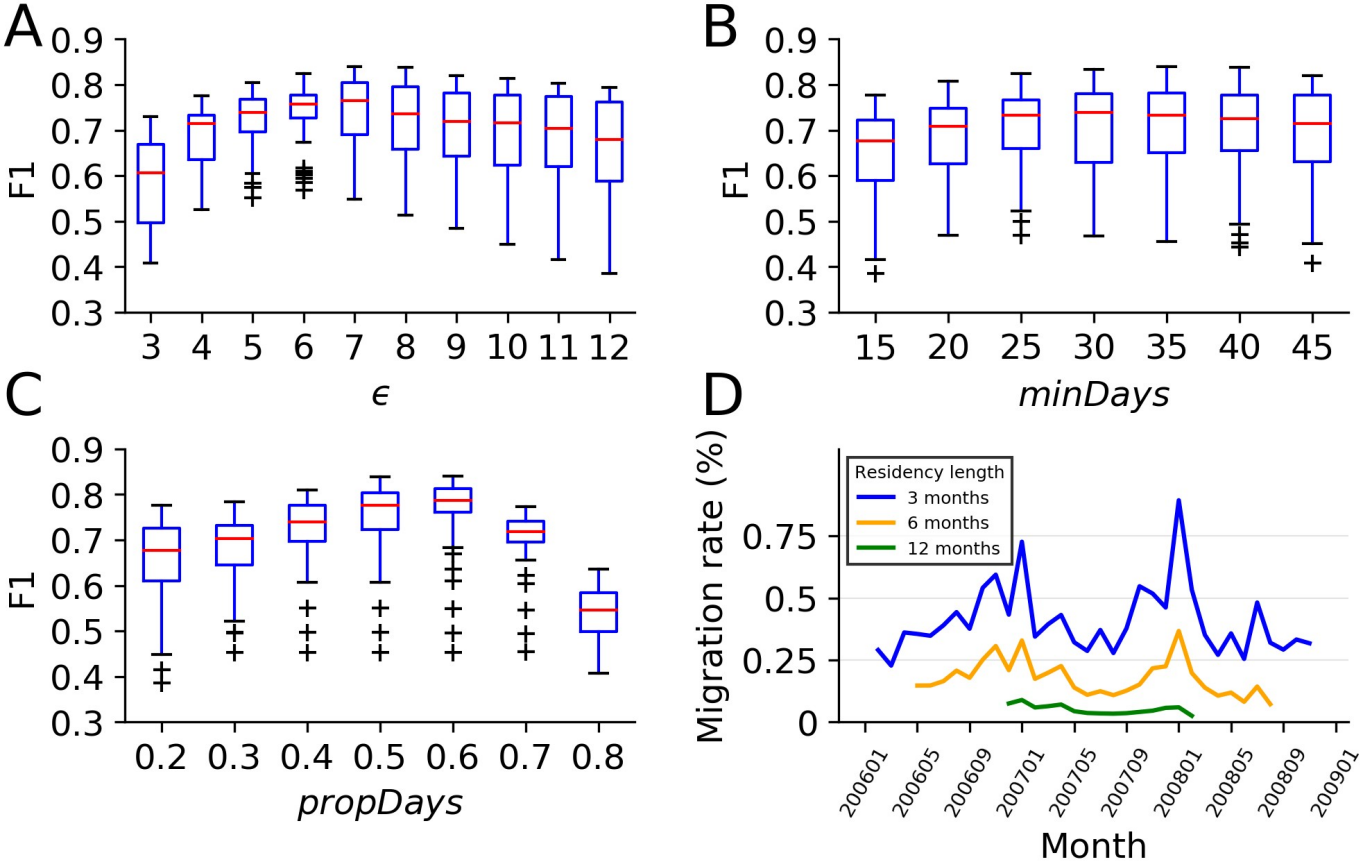
The effect of parameters on performance. (A) Maximum gap between consecutive days. (B) Minimum number of days in a segment. (C) Minimum proportion of days in a segment. (D) Migration rate over time in Rwanda. The optimal tuning of these three parameters is *ϵ* = 7, *minDays* = 30, and *propDays* = 0.6 in our sample.

To understand how the key algorithm parameters impact the resulting migration classifications, we show the impact different parameter values have on the F1 score, based on the sample with human labels. F1 provides a balanced measure of recall and precision. Our approach contains three parameters: the maximum gap between consecutive days *ϵ*, the minimum number of days in a segment *minDays*, and the minimum proportion of days in a segment *propDays*. The relationship between each of these parameters and algorithm performance is shown in Fig 6A, 6B and 6C.

Small values of *ϵ* overlook cases where a person does not appear in the home location for a few days. But large *ϵ* generates very long segments, which can lead to substantial overlap with other segments. When *minDays* is large, it requires a longer sequence of consecutive sightings at the same location, which in turn decreases the number of detected migration events. But if *minDays* is too small, more segments with a long overlap will be found, decreasing the number of detected migration events. The effect of *propDays*, the proportion of appeared days in segments, is similar to *minDays*.

To find the best parameters, we use cross-validation. Specifically, we use five-fold cross validation to split each of the four groups into training dataset (80%) and testing dataset (20%). The best combination of parameters is calculated by using the training dataset to maximize F1 score. We then test the accuracy using this combination of parameters in the testing dataset. We find that the average F1 score of the training dataset is 0.84 and the average F1 score of the testing dataset is 0.83. In our sample, based on the performance on the labeled dataset, the optimal tuning of these three parameters is *ϵ* = 7, *minDays* = 30, and *propDays* = 0.6. Of course, different contents may dictate a different optimal combination.

In settings that are qualitatively different than ours, one would ideally tune these parameters through cross-validation, for instance hand-labeling a sample in order to produce diagnostics similar to those in Fig 6. The need to tune the model‘s hyperparameters is the first limitation of our algorithm. But without such labels, it is still possible to tune the main parameters by observing the impact of different parameter combinations on trajectory maps as those shown in Fig 4. As a general rule of thumb, larger values of *minDays* are useful for detecting long-term migration (to avoid including short-term displacement), whereas small values detect rapid moves. *ϵ* and *propDays* will depend on the frequency and volume of trace data for each individual—if individuals appear almost every day, smaller *ϵ* and larger *propDays* are appropriate.

The second limitation is that the algorithm might not be able to detect continuous segments if we use a large maximum gap between consecutive days *ϵ*, especially for those who do not have phone activities frequently (see S3 Fig for an example). The parameter of the minimum proportion of days in a segment *propDays* has the same issue. This limitation is confirmed when we investigate the samples that have correct labels by the frequency-based approach, but wrong labels by the segment-based approach. We find that the most common reason is that the proportion of days in a segment (*propDays*) is so low that our algorithm does not treat it as a valid segment. While for traditional frequency-based algorithms, they do not measure the proportion of days each month so that they still calculate the modal location each month.

Finally, it is worth noting that in addition to the main parameters of the model, perhaps the most important ‘hyper-parameter’ is *k*, the minimum time an individual must reside in one location to be considered a resident, as discussed at the beginning of the method section. Different values of *k* allow for different types of short- and long-term migrations to be counted. As expected, when migration requires a longer residency length, fewer migrants are detected. This effect is evident in Fig 6D, which shows the migration rate implied by different thresholds of *k* = 3 months, 6 months, and one year. Also evident in the figure is a seasonal migration pattern in January every year, a time when the migration rates almost double.

**Fig 7:**
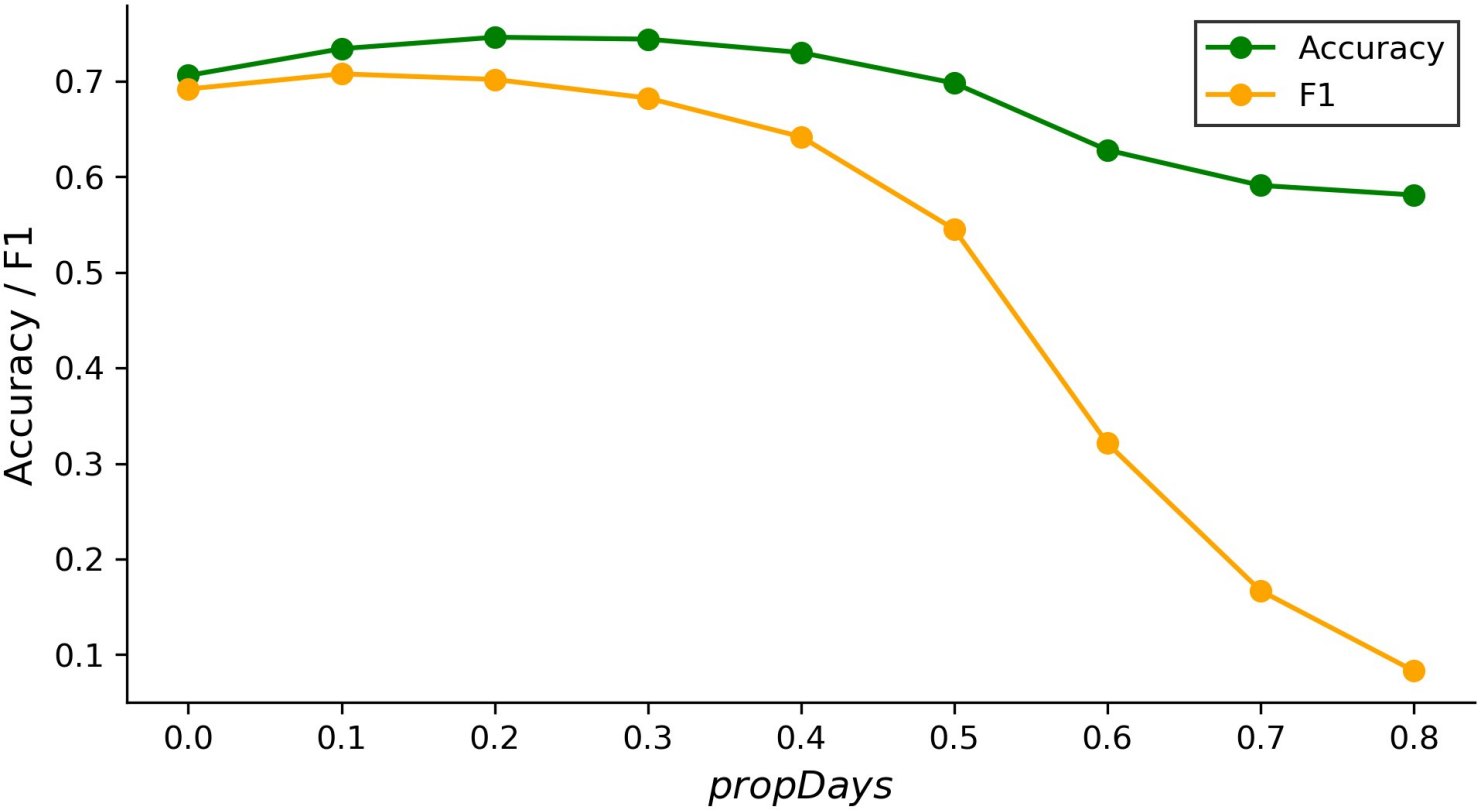
The effect of *propDays* on the performance of frequency-based methods.

The traditional frequency-based method does not contain any ‘parameter’ aside from the length of time in which a person must remain in one place to be considered a resident in that location. However, to better illustrate how the frequency-based approach is impacted by the proportion of appeared days in a month, Fig 7 shows how accuracy varies with *propDays*. We note that the accuracy of the frequency-based method increases slightly from 70.6% to 74.6% as *propDays* increases from 0% to 20%, but the maximum accuracy is still 10 percentage points lower than the algorithm we propose.

## Conclusions

With the increasing prominence and ubiquity of large-scale digital trace data, new opportunities are emerging to study human migration. While a handful of studies have demonstrated this potential, no prior work has carefully considered or validated the computational methods used to infer human migration from these new sources of ‘big’ data. The most common ‘frequency-based’ approach to inferring migration events often results in mis-classifications.

This paper developed a new segment-based approach to measuring migration, and carefully validated the method using a large corpus of migration data labeled by humans. In addition to more accurately classifying migrations, the segment-based approach makes it possible to identify the exact date of migration, and attaches a measure of confidence to each migration event. We have packaged the algorithm in an open-source Python library that is available for public use and modification on GitHub.

While we believe this work represents an important step for researchers using new sources of data to study migration, our hope is that future researchers will continue to adapt our algorithm to other data and contexts. Indeed, beyond the specific segment-based algorithm we have developed, a broader contribution of this paper is to describe a rigorous quantitative framework for evaluating new methods that detect migration from trace data. In particular, by providing new techniques for visualizing individual location trajectories over time, and by showing how human judges can be used to label and cross-validate algorithmic classifications, we hope to lay the groundwork for researchers to rigorously document new algorithms that out-perform our own.

## Supporting information

S1 Fig An example of labeling tasks. Each row is one district in Rwanda. Each column is one day. Labelers are required to answer several questions. For example, whether a migration took place and how confident they are in that assessment on a scale of 1 to 3.
(TIF)

S2 Fig An ambiguous example. Labelers have different opinions on whether a migration event took place in this sample.
(TIF)

S3 Fig An example with a large gap between consecutive days. For those who do not have phone activities frequently, the algorithm might not be able to detect continuous segments if a large maximum gap between consecutive days is used.
(TIF)

S1 Algorithm Pseudocode of detecting location segments. (PDF)

S1 Table A full inventory of the prior work using trace data to study migration. [7–11, 18–21, 57].
(PDF)
