# Supplementary figures and images for: A general approach to detecting migration events in digital trace data
Source: PLoS One. 2020 Oct 2;15(10):e0239408. doi: 10.1371/journal.pone.0239408 (PMC7531812; doi:10.1371/journal.pone.0239408)

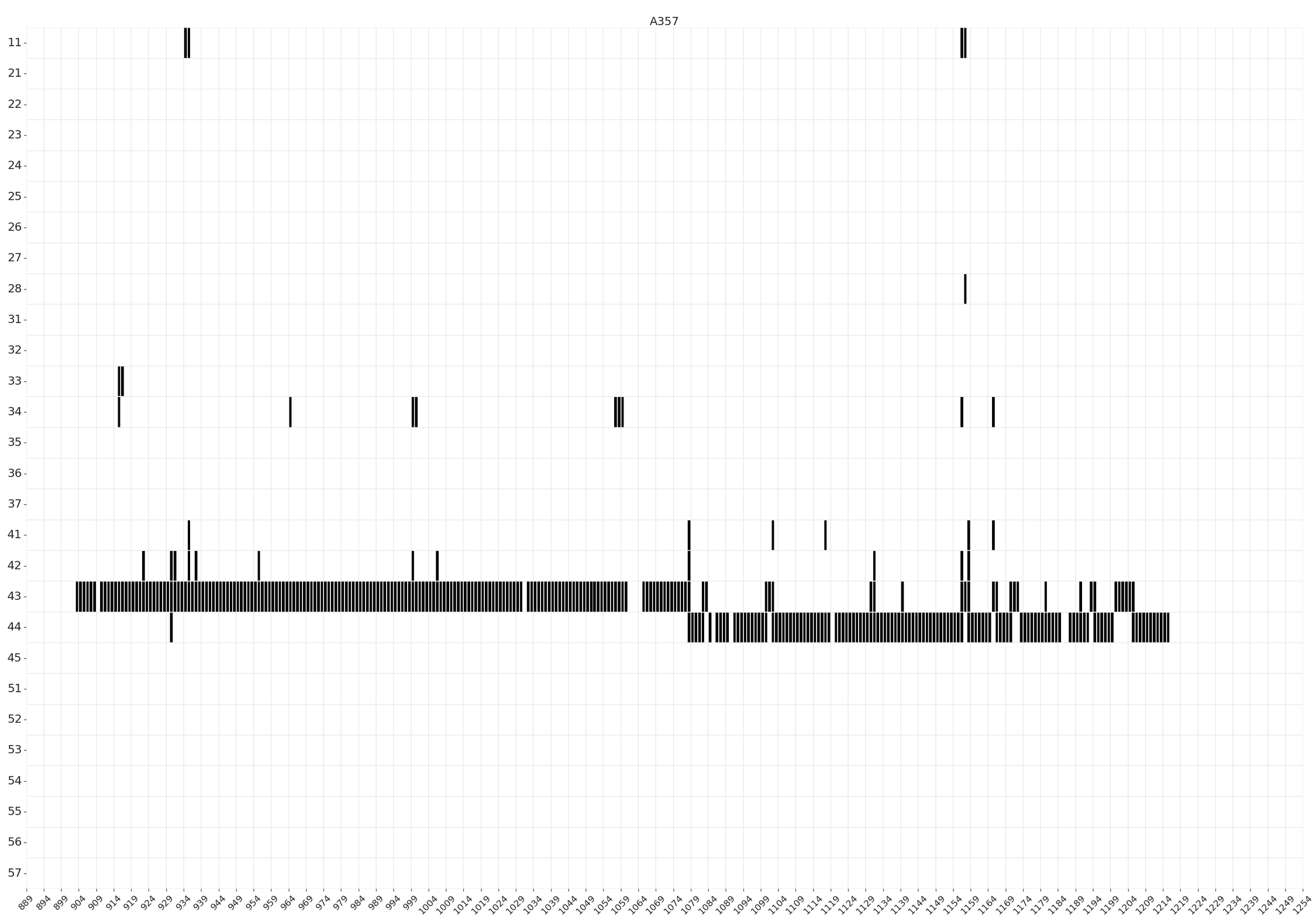

Supplement: S1 Fig — Each row is one district in Rwanda. Each column is one day. Labelers are required to answer several questions. For example, whether a migration took place and how confident they are in that assessment on a scale of 1 to 3. (TIF) [file pone.0239408.s001.tif]

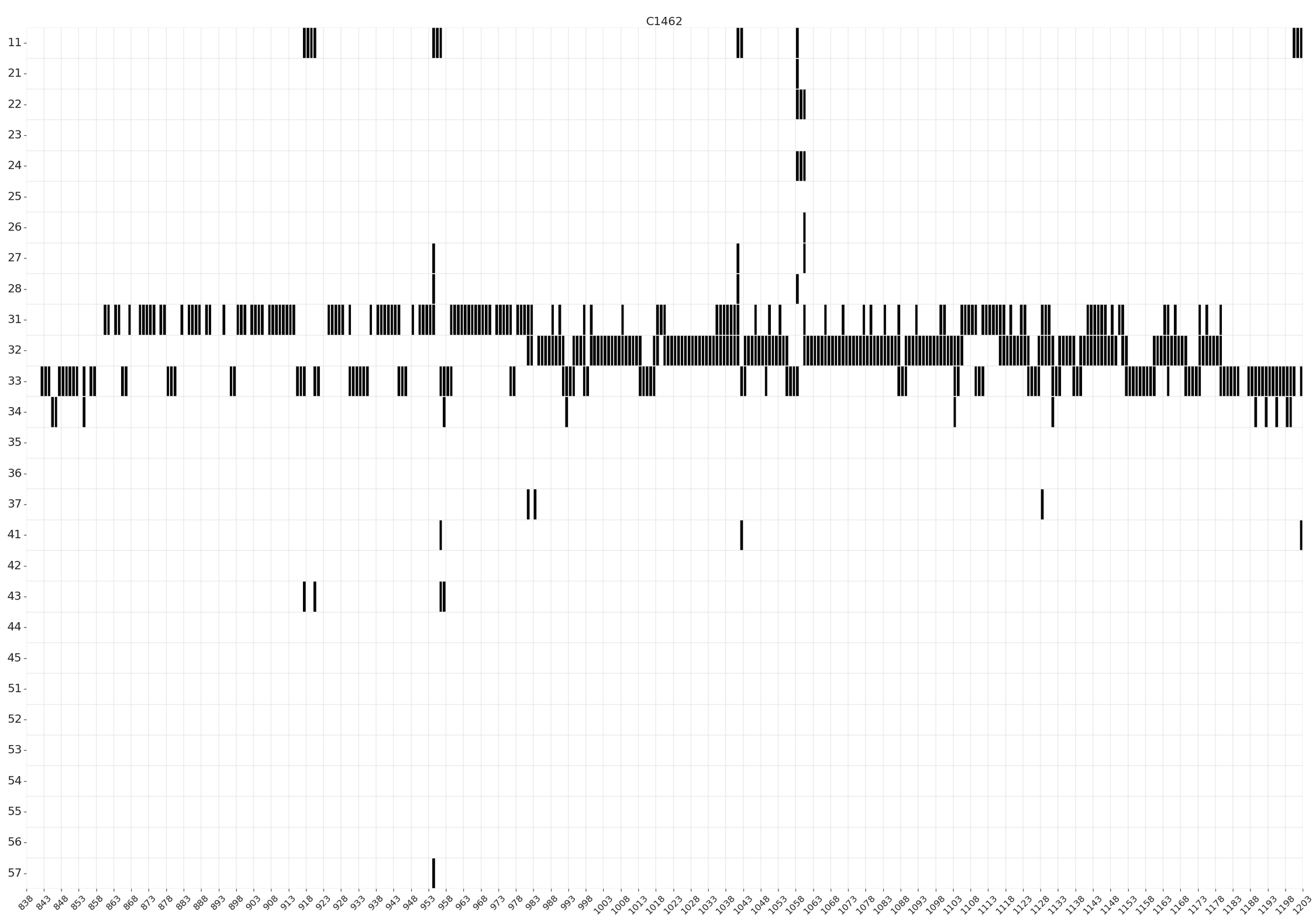

Supplement: S2 Fig — Labelers have different opinions on whether a migration event took place in this sample. (TIF) [file pone.0239408.s002.tif]

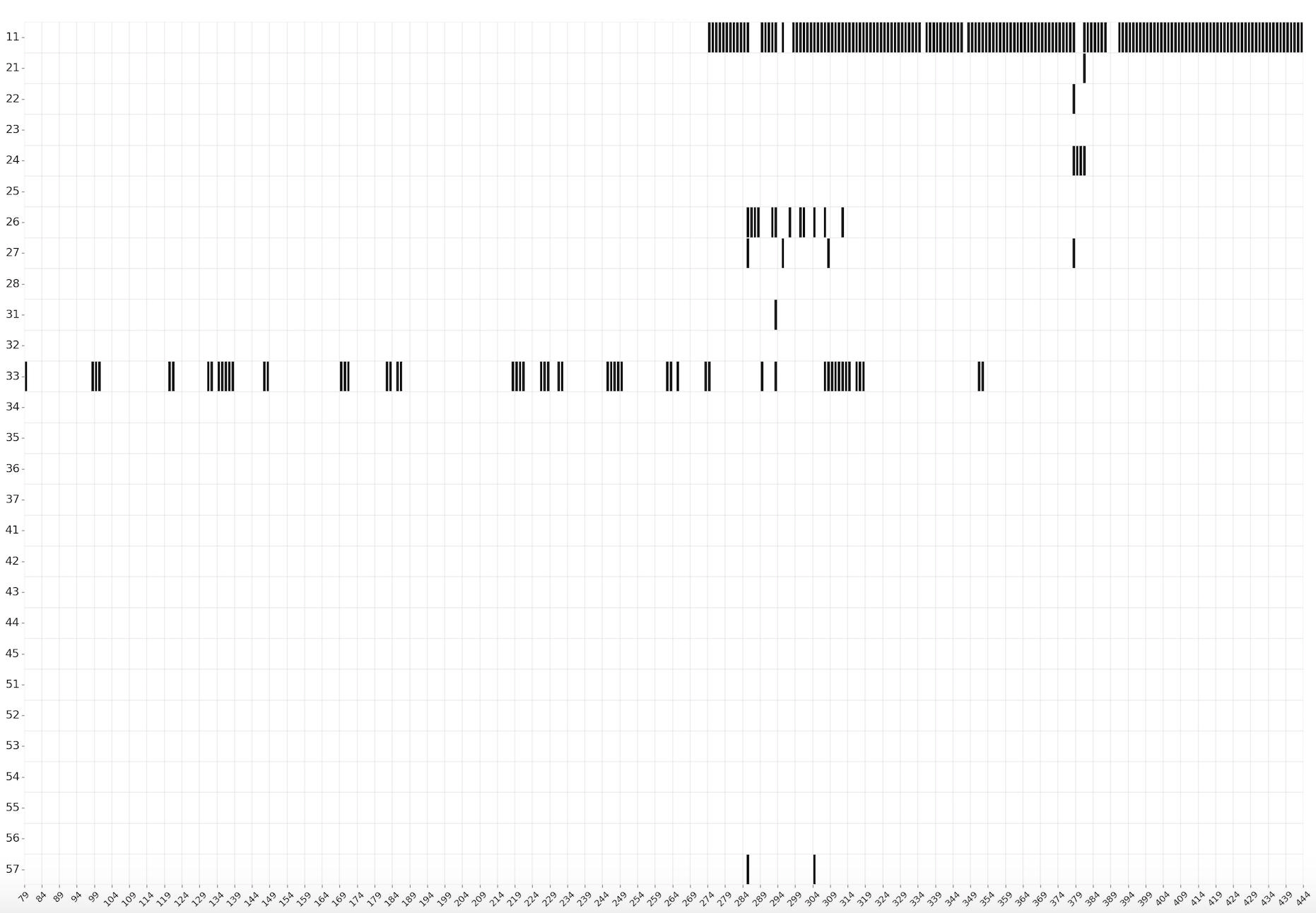

Supplement: S3 Fig — For those who do not have phone activities frequently, the algorithm might not be able to detect continuous segments if a large maximum gap between consecutive days is used. (TIF) [file pone.0239408.s003.tif]
