# Supplementary material for: A general approach to detecting migration events in digital trace data
Source: PLoS One. 2020 Oct 2;15(10):e0239408. doi: 10.1371/journal.pone.0239408 (PMC7531812; doi:10.1371/journal.pone.0239408)
Supplement: S1 Algorithm — (PDF) [file pone.0239408.s004.pdf]

Data: $< userID, timestamp, location >$ tuples for each location record. *Note:* For each individual i, his or her location history can be coded into a matrix M_{ld}, where l is the location among all the locations L; d is the date. l can be a city, or a country, which is determined by the definition of location. $M_{ld} = 1$ if this person appears in the location l in the day d. Otherwise, $M_{ld} = 0$.

Result: $< Segments >$ tuples of users' segments

for $i \in U$ **do**
- **for** $l \in L$ **do**
  - `// Find close days within` ϵ
  - **for** $d \in D$ **do**
    - **if** $M_{ld} == 1$ & $M_{l(d+\epsilon)} == 1$ **then**
      - $M_{l(d+j)} \leftarrow 1$ where j in range(ϵ)
    - **end**
  - **end**
  - `// Group consecutive days into segments`
  - **for** $d \in D$ **do**
    - **if** $M_{ld} == 1$ *for d in range(k) and* $k \geq minDays$ *and* $\sum M^{raw}_{l(d)} \geq k * propDays$ **then**
      - `// save this segment with start date and end date`
      - $Segmt[i][l] += [d, d+k]$
    - **end**
  - **end**
  - `// Merge neighboring segments together if there are no segments in other locations between neighboring segments`
  - **for** $l \in L$ **do**
    - **for** $s \in Segmt[i][l]$ **do**
      - **if** *no other segments exist within* $(s[t-1][1], s[t][0])$ *in other locations* **then**
        - Merge $s[t-1]$ and $s[t]$
      - **end**
    - **end**
  - **end**
  - `// Remove overlapped part between segments`
  - **for** $l \in L$ **do**
    - **for** $s \in Segmt[i][l]$ **do**
      - `// After removing the overlapped part, the end date of the segment` $s[t-1]$ `will be` $s[t][0]-1$`; the first date of the segment` $s[t]$ `will be` $s[t-1][1]+1$`.`
      - **if** *overlap exist:* $(s[t-1][1] > s[t][0])$ **then**
        - $s[t-1][1] = s[t][0] - 1$ $s[t][0] = s[t-1][1] + 1$
      - **end**
    - **end**
  - **end**
- **end**

end

Algorithm 1: Pseudocode of detecting location segments
