# Supplementary material for: A general approach to detecting migration events in digital trace data
Source: PLoS One. 2020 Oct 2;15(10):e0239408. doi: 10.1371/journal.pone.0239408 (PMC7531812; doi:10.1371/journal.pone.0239408)
Supplement: S1 Table — [7–11, 18–21, 57]. (PDF) [file pone.0239408.s005.pdf]

Table S1: A full inventory of the prior work using trace data to study migration.

Paper	Data	Method to identify home	Method to identify migrants
Phithakkitnukoon et al. [19]	CDR	Cell tower where the user has the most call activities (10pm to 7pm) each month	Whose home location changed only once with migration distance of more than 50km
Blumenstock [7]	CDR	Monthly center of gravity (COG) whose distance to COG of last month is smaller than a proportion of average radius of gyration (ROG) over a certain number of months.	Whose migration distance is greater than a proportion of ROG
Lu et al. [20]	CDR	Monthly modal location where the user has the most call activities	Whose home location changed after disasters
Hong et al. [9]	CDR	Monthly modal location where at least 70% of days appear in each of the three consecutive months. Daily modal cell tower is identified as the location where the user has the most call activities (6pm to 6am Monday through Thursday).	Whose home location changed only once
Blumenstock, Chi and Tan [10]	CDR	Same monthly modal location in two/three consecutive months, which is calculated based on daily modal location and hourly modal location. Modal location is where the user has the most call activities.	Whose home location changed
Hankaew et al. [21]	CDR	Same to Phithakkitnukoon et al. [19]	Whose home location changed only once (One home location in two months and a new home location in another two months)
Yang et al. [57]	CDR, plus national ID of each user	Birthplace is extracted from national ID; Living city is the location where the phone number was obtained.	Whose birthplace is different from the living city
Büchel et al. [11]	CDR, plus billing address	Postcode of the billing address	Whose home location changed
Zagheni et al. [8]	Geo-tagged tweets	Modal country of the user over four months (the modal country should has three times more tweets than the second most frequent country)	Whose home location changed
Fiorio et al. [18]	Geo-tagged tweets	Modal tweet location (US county) of the user over a specific duration (duration is a threshold)	Whose home location changed over an interval (interval is another threshold)
